# Current Strategies of Endocrine Therapy in Elderly Patients with Breast Cancer

**DOI:** 10.1155/2018/6074808

**Published:** 2018-01-17

**Authors:** Suk-young Lee, Jae Hong Seo

**Affiliations:** Division of Oncology/Hematology, Department of Internal Medicine, College of Medicine, Korea University, 148 Gurodong-ro, Guro-gu, Seoul 08308, Republic of Korea

## Abstract

Currently, the growing population of the elderly is one of biggest problems in terms of increase in geriatric diseases. Lack of data from large prospective studies on geriatric breast cancer patients often makes it difficult for clinicians to make treatments decisions for them. Because both benefit and risk of treatment should be taken into account, treatment is usually determined considering life expectancy or comorbidities in elderly patients. Treatment of breast cancer is differentiated according to histologic classifications, and hormone therapy is even adopted for patients with metastatic breast cancer if tumor tissue expresses hormone receptors. Endocrine therapy can offer great benefit to elderly patients considering its equivalent efficacy to chemotherapy with fewer toxicities if it is appropriately used. Aromatase inhibitors are usually prescribed agents in hormone therapy for elderly breast cancer patients due to their physiology after menopause. Here, endocrine therapy for elderly patients with breast cancer in neoadjuvant, adjuvant, and palliative setting is reviewed along with predictive adverse events resulting from the use of hormone agents.

## 1. Introduction

With increasing life expectancy, a growing number of patients with geriatric diseases including cancers have become issues of public health. Approximately more than 30% of patients with breast cancer are diagnosed at age over 70 years, and recent data suggest continuous increase of breast cancer incidence rates in women in their 60s (1.0% per year since 2004) and women older than 70 years (1.2% per year since 2005) [1]. Breast cancer is the most frequently diagnosed cancer in the world, and breast cancer alone is expected to occupy 30% of all cancers newly diagnosed in women in 2017 [2]. As the population of elders is expected to grow continuously, 20% of the population is estimated to be at age of 65 years or more by 2030 [3], which will lead to increased number of elderly patients with breast cancer. Despite anticipated increase of geriatric population with breast cancer, large prospective studies on older patients with breast cancer have been scarcely performed. Recent data on breast cancer statistics have shown an improvement of long-term mortality from breast cancer in all age groups from 1989 to 2012 largely due to progress in treatment and early detection through screening [1, 4], but the range of improvement is much smaller in elderly women aged over 70 years (1.5% per year) than that in young women aged 20 to 39 years (2.8% per year) [1]. The narrow range of reduction in mortality paradoxically suggests the lack of screening and the lack of treatment development for elderly breast cancer patients.

Comprehensive assessment on elderly patients, including life expectancy, comorbidities, and performance status, is always necessary to predict the benefit and risk of chemotherapy. While aging is one of reasons for undertreatment using surgery, radiation therapy, or chemotherapy, it is also a cause of increased use of endocrine therapy. Because chemotherapy, which usually accompanies unwanted adverse events, is not always the correct treatment option for patients with breast cancer, decision for initiation of anticancer therapy in geriatric patients with breast cancer might be a little easier if their histologic classifications are clear. Breast cancer expressing hormone receptors is one of candidates that can be managed without toxic chemotherapy. Breast cancer patients with positive expression of estrogen receptor (ER) or progesterone receptor (PgR) without expression of HER2 receptor are good candidates for endocrine therapy with fewer toxicities but equivalent treatment outcomes to chemotherapy. For physiologic reason of menopausal women, aromatase inhibitors (AIs) are often chosen as endocrine therapy agents in geriatric patients with breast cancer expressing hormone receptors. Here, we reviewed the hormone therapy as neoadjuvant, adjuvant, and palliative therapy in elderly patients with breast cancer.

## 2. Management of Geriatric Patients with Malignancies

Because treatment-related complications such as chemotherapy-induced toxicities have been thought to be associated with comorbidities in elderly cancer patients and risk of breast cancer-related mortality is regarded to be relatively reduced in elderly patients due to the elevated risk from other comorbidities-related deaths [22, 23], choice of aggressive treatment is not easy for elderly patients with breast cancer. Results from a previous Surveillance, Epidemiology, and End Results (SEER) registry data analysis assessing probabilities of death from breast cancer in the presence of competing risks showed patients with metastatic disease aged 70 years or older died from breast cancer, but causes of mortality in patients with other stages were attributed to comorbid diseases [24]. An observational study using four nationwide population registries in Denmark also reported that mild to moderate comorbidity assessed by Charlson Comorbidity Index affects mortality of breast cancer patients aged 50–79 years receiving chemotherapy [25]. An agreement exists that chronologic age itself should not be the only determinant in treatment of elderly patients with cancers. For the heterogeneity of the elderly in the same age in terms of physical, psychological, and cognitive function, as well as financial and social status, biological age taking consideration into individual health status and comorbid disease should be taken account in treatment decision. In order to address health status objectively, the International Society of Geriatric Oncology and the National Comprehensive Cancer Network recommend comprehensive geriatric assessment (CGA) before treatment decisions [26, 27]. CGA is a systematic procedure to assess multiple comorbidities and functional status of old patients through which geriatric problems not detected by routine oncology approach can be found. Several studies have reported that components of CGA, comorbid diseases, functional status, cognitive function, nutritional status, geriatric syndromes, and polypharmacy, are associated with survival and toxicity of chemotherapy in older patients with malignancies [28, 29]. In breast cancer, a study with patients older than 65-year harboring stage 1-3A has reported that the proportion of women who survived 10 years is significantly decreased as number of cancer-specific geriatric assessment (C-SGA) deficits is increased (linear trend *p* < 0.0001) [28]. However, despite the proven benefit of CGA, this time-consuming tool limits its application to the busy clinical practice. Several screening tools such as Abbreviated CGA (aCGA), Geriatric 8 (G-8), Vulnerable Elders Survey (VES-13), Triage Risk Screening Tool (TRST 1+), and Groningen Fraility Index have been developed to overcome this disadvantage. Shorter and comprehensive geriatric evaluation is possible with these screening tools, but they should not replace CGA due to the lack of data that support the predictability of those screening tools for outcomes of CGA. A current guideline recommends the use of these screening tools only for the identification of patients who would benefit from CGA [27, 30].

## 3. Tumor Biology of Elderly Breast Cancer

Biology and pathologic characteristics of breast cancer seem to change with increasing age. It is generally known that tumor biology of older patients with breast cancer shows less aggressive and indolent features, decreased frequency of axillary lymph node metastasis, vascular invasion, and lymphoplasmacytic stromal reaction [31]. Results from a retrospective study of 1758 women older than 70 years comparing tumor biology with younger counterparts showed higher expression of ER, PgR, Bcl2, and Muc1 but low expression of HER2, Ki76, p53, and EGFR in older group [32]. Moreover, the proportion of ER-positive breast cancer is increased with increasing age in cohort of patients over 65 years according to San Antonio Breast Cancer Database and SEER. It was revealed that 87% of patients aged 65 to 74 years showed ER positivity in their tumors, and the proportion of ER positivity was increased to 91% in patients older than 85 years [33]. Contrary to ER, HER2 expression is known to be less frequent in elderly patients with breast cancer; the proportion of cases with HER2 positive tumors is 4% in the cohort of over-60 years, while it is 9% in patients younger than 35 years [30, 34]. These results might mislead clinicians to conclude that endocrine therapy is the best choice in treatment of breast cancer of the elderly; however, it is known that the efficacy of hormone therapy is not correlated with age [35, 36].

On the other hand, some studies report aggressiveness of breast cancer in the elderly. A retrospective analysis from a single-institution showed greater likelihood of distant metastases in a subgroup of elderly patients aged over 70 years compared to that in the younger patients [37]. In addition, another study also reported that breast cancer was often detected at far advanced stage in the elderly; only 47% of patients with breast cancer aged over 75 years were found to have T1 tumors, while 70% of patients aged between 45 and 64 years were found to have T1 tumor, probably due to the lack of screening or delayed diagnosis [38–40].

Relevance of histologic classification of breast cancer to age has also been suggested by a retrospective analysis showing less aggressive features in the elderly in some cases. Certain histologic subtypes including infiltrating lobular carcinomas, mucinous carcinomas, and papillary carcinomas, which are slowly proliferating and low-grade tumors, have shown a gradual rise in incidence with increasing age [30, 31].

## 4. Endocrine Therapy in Elderly Breast Cancer Patients

Because little data are available on endocrine therapy for elderly patients with breast cancer, these patients are generally treated as postmenopausal women. After menopause, adrenal glands and adipose tissue take over the role as major estrogen producing organs from ovaries. The fact that aromatase is an enzyme that acts on conversion of testosterone to estradiol and androstenedione to estrone, major source of estrogen in postmenopausal women [41], makes aromatase inhibitors (AIs) be preferred agents of hormone therapy for postmenopausal women with breast cancer. All currently used AIs are third generation represented by letrozole, anastrozole, and exemestane, exerting more potent efficacy comparing to first and second generations of AIs. Third generation AIs are classified as steroidal (type 1) and nonsteroidal (type 2) types according to their mechanisms of action (Table 1). Type 1 AI, exemestane, inactivates aromatase by irreversibly binding to substrate's binding site of the enzyme. Type 2 AIs are letrozole and anastrozole which inhibit aromatase reversibly by binding to haem moiety of the enzyme [42, 43]. Agents used in endocrine therapy of elderly breast cancer patients are summarized in Table 2.

### 4.1. Hormone Therapy in Neoadjuvant Treatment

The efficacy of AIs in downstaging and reducing tumor volume before surgical interventions in postmenopausal women with breast cancer positive for hormone receptors who are potentially operable has been demonstrated in several randomized studies (Table 3) [5–8]. Two prospective phase 2 studies compared the efficacy of neoadjuvant therapy by AIs to chemotherapy in postmenopausal women with hormone receptor-positive breast cancer. One study randomized ER-positive patients to receive anastrozole or exemestane for three months or doxorubicin and paclitaxel. Neoadjuvant endocrine therapy showed equivalent efficacy to chemotherapy in terms of overall objective response, pathologic complete response, and rates of breast-conserving surgery in ER-positive postmenopausal women [9]. Another study randomized postmenopausal or premenopausal patients with luminal breast cancer to receive chemotherapy (epirubicin plus cyclophosphamide followed by docetaxel) or hormone therapy with exemestane. No significant difference in clinical response rate was observed, suggesting equivalent efficacy of neoadjuvant endocrine therapy to chemotherapy [10].

### 4.2. Hormone Therapy as Adjuvant Treatment

The efficacy of AIs in adjuvant setting has been examined in several studies (Table 4). Anastrozole Tamoxifen Alone and in Combination (ATAC) trial was a head-to-head trial comparing efficacy of anastrozole, an AI, with tamoxifen. More than 9000 postmenopausal patients were recruited, and 3125 were randomized to have anastrozole, 3116 to take tamoxifen, and 3125 to use a combination of both agents. The ATAC trial demonstrated that anastrozole was superior in terms of disease-free survival (DFS), distant metastases, and contralateral breast cancer compared to tamoxifen in postmenopausal women with early stage breast cancer [11, 43–45]. DFS at three years was 89.4% in the anastrozole group and 87.4% in the tamoxifen group (hazard ratio (HR) 0.83, 95% confidence interval (CI) 0.71–0.96, *p* = 0.013), and the superiority was maintained at 10 years (HR 0.91, 95% CI 0.83–0.99, *p* = 0.04). The favorable result of anastrozole over tamoxifen in DFS was more prominent in hormone receptor-positive postmenopausal breast cancer patients (HR 0.86, 95% CI 0.78–0.95, *p* = 0.003). After a median follow-up of 68 months, incidence of contralateral breast cancer (42% reduction, 95% CI 12–62, *p* = 0.01) and distant metastases (HR 0.86, 95% CI 0.74–0.99, *p* = 0.04) were significantly lower in the anastrozole group than in the tamoxifen group [11, 44, 45]. Furthermore, anastrozole appeared to be more beneficial for postmenopausal women aged more than 65 years in a subgroup analysis (HR 1.19, 95% CI 1.04–1.36) [44]. The combination therapy with tamoxifen and anastrozole had no advantage over tamoxifen monotherapy in DFS (HR 1.02, 95% CI 0.89–1.18, *p* = 0.8).

The Breast International Group (BIG) 1-98 is a randomized phase 3 trial comparing two different groups receiving letrozole first with two different groups receiving tamoxifen initially [12, 46]. The two letrozole initial groups were letrozole alone for 5 years and letrozole 2 years followed by tamoxifen for 3 years. The two tamoxifen first groups were tamoxifen monotherapy for 5 years and tamoxifen for 2 years followed by letrozole for 3 years. DFS was superior in the letrozole initial group to the tamoxifen first group (HR 0.81, 95% CI 0.70–0.93, *p* = 0.003) with a median follow-up of 25.8 months. Initial use of letrozole was also more beneficial in elderly patients aged more than 65 years in terms of DFS (HR 0.79, 95% CI 0.64–0.97, *p* = 0.02) [46]. With a longer follow-up, a median follow-up of 71 months, updated results showed no significant difference in DFS between letrozole monotherapy group and either sequential treatment groups (HR for tamoxifen and letrozole sequential group 1.05, 99% CI 0.84–1.32; HR for letrozole and tamoxifen sequential group 0.96, 99% CI 0.76–1.21) [12].

A letrozole extension study with large numbers of elderly breast cancer patients, the MA. 17 trial, showed benefit of extended therapy with letrozole after completion of adjuvant tamoxifen in postmenopausal women with hormone receptor-positive early stage breast cancer [13]. A total of 5,187 receptor-positive, postmenopausal early breast cancer patients who were disease-free after 5 years of treatment with tamoxifen were randomly assigned to receive either letrozole or placebo. Improved DFS was demonstrated in letrozole treated patients (HR, 0.58, 95% CI 0.45–0.76, *p* ≤ 0.001) at a median follow-up of 30 months. Patients were further subdivided into three age groups (<60 years, 60 to 69 years, and ≥70 years) to see benefits of letrozole in elderly patients. Significantly different DFS favoring letrozole was shown only in patients younger than 60 years (HR 0.46, *p* ≤ 0.001). Because there was no interaction between age and treatment, the study suggested consideration of extended adjuvant therapy with letrozole after completing 5-year tamoxifen treatment in patients older than 70 years [47].

Two separate meta-analyses also demonstrated the superiority of AIs. Results from a meta-analysis of individual data on 31920 postmenopausal, ER-positive early stage breast cancer patients revealed the superiority of AIs to tamoxifen by showing reduced recurrence rate and decreased 10-year breast cancer mortality [48]. Another study by meta-analysis also proved efficacy of AIs with significantly lower recurrence rates compared to tamoxifen. There was no obvious heterogeneity between all age-subgroups. There was a 22% reduction in recurrence (SE 0.10) in patients aged over 70 years in the initial monotherapy with AIs group and a 19% reduction (SE 0.13) in those who used AIs after 2-3 years of tamoxifen comparing to tamoxifen monotherapy [49].

### 4.3. Hormone Therapy as Palliative Anticancer Treatment

Considering great benefits with relatively fewer toxicities in association with hormone therapy, endocrine therapy is the most appropriate treatment modality for elderly breast patients. Patients who can benefit from endocrine therapy should always be accessed, and postmenopausal women with hormone receptor-positive metastatic or recurrent breast cancer are candidates for endocrine therapy. Clinical trials of endocrine therapy in palliative setting are summarized in Table 5.

#### 4.3.1. First-Line Endocrine Therapy in Postmenopausal Patients with Metastatic Breast Cancer

Several randomized phase 3 studies have demonstrated at least equivalence or the superiority of anastrozole, letrozole, and exemestane over tamoxifen in postmenopausal women with metastatic breast cancer (Table 5) [14–17, 50, 51].

A series of recent studies have raised palbociclib (PD-0332991), a reversible and selective inhibitor of cyclin dependent kinases 4 and 6 (CDK 4/6), as one of agents recommendable for first-line and salvage endocrine therapy. CDK4 and CDK6, known to be activated by cyclin D, regulate G1-S transition of cell cycle by hyperphosphorylation of Rb [52]. Many experimental data have suggested that inhibition of cyclin D activity may lead to suppression of tumor growth and tumor cell death [53, 54]. The inhibitory effect of PD-0332991 was also shown in breast cancer cell lines, especially in luminal ER-positive human breast cancer cell lines including those with HER2 amplification. Meanwhile, nonluminal/basal breast cancer subtypes were found to be resistant to the inhibitory effect of PD-0332991 [55]. Based on those findings, clinical studies have investigated the efficacy of palbociclib in ER-positive postmenopausal advanced breast cancer patients [18, 56]. Results of a phase 2, open-label, randomized study investigating the efficacy of palbociclib in combination with letrozole compared to letrozole alone as first-line therapy in postmenopausal women with advanced ER-positive and HER2-negative breast cancer (PALOMA-1/TRIO-18) were reported about 2 years ago. The combination group showed two times of improvement in median progression-free survival (PFS) compared to the letrozole alone group (median PFS 20.2 versus 10.2 months, HR 0.488, 95% CI 0.319–0.748) [18].

Fulvestrant is a 17*β*-estradiol analog that inhibits estrogen signaling by downregulating the expression of ER protein [57]. Fulvestrant First-Line Study Comparing Endocrine Treatments (FIRST) is a phase 2, randomized, open-label, multicenter trial investigating the efficacy of fulvestrant 500 mg compared to anastrozole 1 mg in postmenopausal ER-positive patients with advanced breast cancer who had no previous treatment. Results from this clinical trial showed equivalent efficacy of fulvestrant to anastrozole in terms of clinical benefit rate defined as the proportion of patients with objective response or stable disease for ≥24 weeks (72.5% versus 67%, OR 1.3, 95% CI 0.72–2.38, *p* = 0.386) and overall response (36.0% versus 35.5%, OR 1.02, 95% CI 0.56–1.87, *p* = 0.947). Time to progression (TTP) (median TTP 23.4 versus 13.1 months, HR 0.66, 95% CI 0.47–0.92, *p* = 0.01) and overall survival (OS) (median OS 54.1 versus 48.4 months, HR 0.7, *p* = 0.04) were shown to be improved in the fulvestrant group compared to the anastrozole group [19, 58, 59]. A prospective phase 3 clinical trial is currently undergone (NCT01602380).

#### 4.3.2. Endocrine Therapy Beyond Progression to First-Line Hormone Treatment in Postmenopausal Women with Metastatic or Recurrent Breast Cancer

Resistance to hormone therapy in patients with receptor-positive breast cancer is not unusual. One of suggested resistance mechanisms to endocrine therapy is aberrant activation of phosphatidylinositol 3-kinase- (PI3k-) Akt-mammalian target of rapamycin (mTOR) signaling pathway [60–62]. Breast Cancer Trials of Oral Everolimus-2 (BOLERO-2) study is a phase 3 randomized clinical trial that compares everolimus, an mTOR inhibitor, and exemestane versus exemestane and placebo in ER-positive postmenopausal patients with advanced breast cancer refractory to previous letrozole or anastrozole. After a median follow-up of 18 months, median PFS was shown to be significantly longer in the everolimus plus exemestane group than that of the placebo plus exemestane group (median PFS 7.8 versus 3.2 months, HR 0.45, 95% CI 0.38–0.54, *p* < 0.0001) [20, 63]. Analysis on elderly patients aged 65 years or more was performed among patients enrolled in the BOLERO-2 study, and significantly improved PFS was consistently reported in those patients (HR in ≥65 years 0.59, 95% CI 0.43–0.80; HR in ≥70 years 0.45, 95% CI 0.30–0.68). Older patients treated by everolimus showed similar incidence of adverse events compared to younger patients, but they had more frequent incidence of on-treatment deaths (on-treatment deaths in those <70 years of age, 1.3%; in those ≥70 years, 7.7% in exemestane plus everolimus group) [64].

A phase 3 study with advanced hormone receptor-positive, HER2 negative breast cancer patients who relapsed or progressed during previous hormone therapy was performed to compare the efficacy of palbociclib and fulvestrant versus placebo and fulvestrant. This study included both pre- and postmenopausal women. The combination therapy showed superior efficacy with longer median PFS (9.2 versus 3.8 months, HR 0.42, 95% CI 0.32–0.56, *p* < 0.001). The majority of patients in this study were postmenopausal women (>70%). HR for disease progression in patients older than 65 years was comparable to that in younger patients (HR in ≥65 years 0.35, 95% CI 0.19–0.62; HR in <65 years 0.44, 95% CI 0.32–0.61, *p* = 0.48) in subgroup analysis [21].

## 5. Adverse Effects of Hormone Therapy

Although AIs are relatively tolerable, adverse effects from their prolonged use should always be considered and managed adequately to improve compliance. Due to their different action of mechanisms, no proven estrogenic effects of AIs, spectrum of adverse effects of AIs is somewhat discriminated from that of selective estrogen receptor modulator (SERM), tamoxifen. Several clinical studies on those hormone agents have reported slightly better toxicity profiles of AIs.

### 5.1. Musculoskeletal Complication

Musculoskeletal complication is one of discriminating adverse events attributing to the use of AIs from the use of SERM. The majority of clinical trials on AIs showed significantly increased incidence of musculoskeletal events in patients taking AIs comparing to those having tamoxifen or placebo [16, 45, 47, 65]. Contrary to the protective effect of tamoxifen on bone loss and bone mineral density [66], AIs are generally known to be associated with osteoporosis and bone fracture [67]. A systematic review of 11 randomized controlled trials (RCTs) on adverse bone outcomes in the elderly using AIs reported that fracture risk of AIs is 1.5 times higher than that of tamoxifen or placebo [68]. In previously conducted ATAC trial, significantly larger numbers of patients with anastrozole were reported to experience skeletal events such as arthralgia and fracture than those with tamoxifen [45, 68]. Data from toxicity analysis of letrozole in MA.17 trial showed patients with letrozole had significantly more frequent incidence of arthralgia than those with placebo in patients younger than 70 years [47]. The fracture rate was not shown to be significantly different between two groups, and this might be attributed to tamoxifen effect which had been used for 5 years before randomization to letrozole [68]. Considering adverse skeletal events attributing to AIs, all patients with AIs are recommended to take advice on exercise, calcium-vitamin D supplements, and bone mineral density (BMD) monitoring. If T-score is less than −2.0 or at least two risk factors for fracture are observed, bisphosphonate therapy should be considered. Patients with T-score more than −2.0 without accompanying risk factors are treated based on BMD loss during 1-2 years. Risk factors for fracture in patients with breast cancer include the use of AIs, T-score < −1.5, age older than 65 years, BMI < 20 kg/m^2^, family history of hip fracture, history of fragility fracture after age 50 years, prolonged corticosteroid use, and smoking [69]. A large quantity of data support bisphosphonate or a RANKL inhibitor, denosumab, therapy as appropriate antiabsorptive therapy to prevent osteoporosis in postmenopausal women having AIs.

Four independent studies on efficacy of zoledronic acid (4 mg i.v. q 6 months) and one study on denosumab (Hormone Ablation Bone Loss Trial in Breast Cancer; HALT-BC, 60 mg s.c. q 6 months) demonstrated their benefits in preventing bone loss [70–74]. More encouraging recent results from a study on adjuvant bisphosphonate therapy are antitumor effect and survival benefit of antiabsorptive therapeutic agent. A meta-analysis from EBCTCG reported that the use of adjuvant bisphosphonate in postmenopausal women reduced recurrence and mortality from breast cancer [75]. All results from these studies support the use of bisphosphonate and denosumab in postmenopausal women treated with AIs, but unwanted adverse events from using these antiabsorptive agents should be noted. Osteonecrosis, renal insufficiency, myalgia, arthralgia, and electrolyte imbalance including hypocalcemia are complications that should be considered before initiating adjuvant bisphosphonate therapy. Long-term risks of denosumab therapy have not been reported yet.

### 5.2. Endometrial Cancer

Avoidance of postmenopausal women with AIs from exposure to estrogen made favorable toxicity profile for them in terms of endometrial cancer, vaginal discharge, and bleeding. The ATAC study reported more frequent incidence of vaginal bleeding and discharge and endometrial cancer in patients having tamoxifen [45]. Five-year administration of tamoxifen before the use of letrozole resulted in equivalent frequency of vaginal bleeding between letrozole and placebo groups in MA.17 study [47]. A meta-analysis of RCTs comparing AIs with tamoxifen also reported reduced risk of AIs for endometrial cancer [Odds ratio (OR) 0.34, 95% CI 0.22–0.53, *p* < 0.001] [76]. Another patient-level meta-analysis of 31920 postmenopausal women also showed lower incidence of endometrial cancer with AIs than tamoxifen (10-year incidence 0.4% versus 1.2%, relative risk 0.33, 95% CI 0.21–0.51). The decreased incidence of endometrial cancer with AIs was independent of age and lasted for years after finishing the endocrine therapy [48].

### 5.3. Vascular Events

Due to the absence of estrogenic effect in AIs, incidence of vascular events including thromboembolic and cerebrovascular events is also expected to be decreased compared to that in patients taking tamoxifen. Results from safety analysis of ATAC trial showed reduced incidence of ischemic cerebrovascular (anastrozole versus tamoxifen 1.1% versus 2.3%, *p* < 0.001), all venous thromboembolic (2.2% versus 3.8%, *p* < 0.001), and deep venous thromboembolic events (1.1% versus 1.8%, *p* = 0.027), although no difference in frequency of ischemic cardiovascular events (2.8% versus 2.2%, *p* = 0.121) was observed [77]. Decreased incidence of thromboembolic events was consistently observed in the BIC 1-98 trial (letrozole versus tamoxifen 1.5% versus 3.5%, *p* < 0.001). Cerebrovascular accident or transient ischemic accident (1.0% versus 1.0%, *p* = 0.91) and ischemic heart disease (1.4% versus 1.2%, *p* = 0.28) showed no significant difference in incidence between letrozole and tamoxifen groups in that study [46]. A meta-analysis of 30023 patients also showed coherent results in terms of adverse vascular events. This study reported no significant difference in the odds of cerebrovascular disease between the two groups (OR 1.01, 95% CI 0.81–1.26, *p* = 0.93); meanwhile it significantly increased odds in AIs users in terms of cardiovascular disease (OR 1.30, 95% CI 1.06–1.61, *p* = 0.01) and decreased odds in venous thrombosis in AIs users (OR 0.55, 95% CI 0.46–0.64, *p* < 0.001) [76].

## 6. Adherence to Endocrine Therapy

Due to less severe toxicity profile of endocrine therapy compared to chemotherapy, it is usually expected that patients will adhere to endocrine therapy. However, results from the MA. 17 trial showed that the proportion of patients who discontinued treatment due to toxicities in the elderly aged over 60 years was similar between letrozole and placebo groups [47]. BIG 1-98 prospective trial showed that the proportions of treatment discontinuation were 38.4% in patients aged 75 years or more and 22.66% in those younger than 75 years without significant difference between letrozole and tamoxifen groups (*p* = 0.71) [78]. These data suggest that age itself other than toxicities might be one of major factors affecting the adherence to endocrine therapy. A systematic review, in which most studies focused on compliance to adjuvant endocrine therapy in patients with breast cancer, evaluating adherence to cancer treatment in elderly patients found that adherence rate varied from 52% to 100%. Factors for nonadherence were shown to vary across studies [79]. Another systematic review on adherence to medications also described various factors such as socioeconomic-related factors, healthcare team related factors, system related factors, condition-related factors, therapy-related factors, and patients-related factors as determinants of patients compliance [80]. As a matter of fact, several studies reported poor adherence and low use of adjuvant endocrine therapy in low-income breast cancer patients [81–83]. It seems that health status represented by presence of comorbidities in addition to chronological age is also an important contributing factor to adherence to endocrine therapy in stage 2 to 3 breast cancer patients. [84]. Duration of endocrine therapy is another factor for adherence. A study on endocrine therapy use in elderly breast cancer patients showed noncompliance with time from diagnosis [81]. Therefore, considering necessity of long-term adherence in endocrine therapy, not only age but also various circumstances that patients on treatment are facing should be taken into account to improve compliance, because improvement in compliance consequently can lead to better treatment outcomes.

## 7. Conclusion

Despite prolongation of life expectancy and subsequent growing number of geriatric population with breast cancer, progress of studies on those patients, a considerable proportion in patients with breast cancer, is below expectations at present. In this review, we summarized representative studies on AIs used in postmenopausal women. We also tried to focus on studies which included subgroup analyses on elderly patients. However, because there are several problems in currently performed studies on geriatric breast cancer patients, limitation also exists in this study.

The lack of large prospective studies in which patients with breast cancer are consisted only with the elderly is one of rush assignments to be solved to provide appropriate guide in treatment of geriatric patients with breast cancer. Exploration on conditions where endocrine therapy can be the most appropriate treatment choice other than known histology subclassification could also be useful given that hormone therapy is a relatively easy remedy to apply to geriatric patients. Although endocrine therapy is usually regarded as treatment with less burden for risks compared to chemotherapy, AI-specific adverse events such as skeletal events should always be monitored by treatment providers. Various situations that decrease adherence to treatment in the elderly are also one of problems we should consider in their management to improve treatment outcomes. Further efforts are needed to find appropriated therapy in older breast cancer patients to prepare for the coming geriatric era in the near future.

## Figures and Tables

**Table 1:** Classification of aromatase inhibitors.

	1st generation	2nd generation	3rd generation
Steroidal (type 1)		Formestane	Exemestane

Nonsteroidal (type 2)	Aminoglutethimide	Fadrozole	Letrozole
			Anastrozole
			Vorozole

**Table 2:** Endocrine therapy of the elderly with breast cancer.

Hormone agent	Mechanism	Dose
Letrozole	Reversible AIs	2.5 mg daily PO
Anastrozole	Reversible AIs	1 mg daily PO
Exemestane	Irreversible AI	25 mg daily PO
Fulvestrant	SERD	500 mg IM q 28 days
Everolimus	mTOR inhibitor	10 mg daily PO
Palbociclib	CDK 4/6 inhibitor	125 mg daily PO

AI, aromatase inhibitor; SERD, selective estrogen receptor downregulator; mTOR, mammalian target of rapamycin; CDK, cyclin dependent kinase.

**Table 3:** Clinical trials of neoadjuvant endocrine therapy in elderly patients with breast cancer.

Study	Design	Number of patients	Treatment	Primary end point	Results	*p*
PROACT [5]	Phase 3, randomized, double-blind	451	Anastrozole 1 mg versus tamoxifen 20 mg	OR	39.5 versus 35.4%; odds ratio 1.24	0.29
IMPACT [6]	Phase 3, randomized, double-blinded	330	Anastrozole 1 mg versus tamoxifen 20 mg versus anastrozole 1 mg + tamoxifen 20 mg	OR	37 versus 36 versus 39%	
Z1031 [7]	Phase 2, randomized	374	Exemestane 25 mg versus letrozole 2.5 mg versus anastrozole 1 mg	OR	62.9 versus 74.8 versus 69.1%	
Eiermann et al. [8]	Phase 3, randomized, double-blinded	337	Letrozole 2.5 mg versus tamoxifen 20 mg	OR	55% versus 36%	<0.001
Semiglazov et al. [9]	Phase 2, randomized	239	Anastrozole 1 mg or exemestane 25 mg versus doxorubicin + paclitaxel	OR	64.5 versus 63.6%	>0.5
GEICAM/2006-03 [10]	Phase 2, randomized	95	Exemestane 25 mg versus EC-T	OR	48 versus 66%	0.075

No, number; OR, objective response; EC-T, epirubicin and cyclophosphamide followed by docetaxel.

**Table 4:** Clinical trials of adjuvant endocrine therapy in elderly patients with breast cancer.

Study	Design	Number of patients	Treatment	Primary end point	Results	*p*
ATAC [11]	Phase 3, randomized	9366	Anastrozole 1 mg versus tamoxifen 20 mg versus anastrozole 1 mg + tamoxifen 20 mg	DFS	A versus T; HR 0.91, 95% CI 0.83–0.99	0.04
BIG 1-98 [12]	Phase 3, randomized, double-blind	8010	T-L versus letrozole L-T versus letrozole	DFS	HR 1.05, 99% CI 0.84–1.32 HR 0.96, 99% CI 0.76–1.21	
MA. 17 [13]	Phase 3, randomized, double-blind	5187	Tamoxifen-letrozole versus tamoxifen-placebo	DFS	HR 0.58, 95% CI 0.45–0.76	<0.001

No, number; DFS, disease-free survival; A, anastrozole; T, tamoxifen; HR, hazard ratio; CI, confidence interval; L, letrozole.

**Table 5:** Clinical trials of palliative endocrine therapy in elderly patients with breast cancer.

Study	Design	Number of patients	Treatment	Primary end point	Results	*p*
*First line*						
TARGET [14]	Phase 3, randomized, double-blind	668	Anastrozole 1 mg versus tamoxifen 20 mg	TTP OR	8.2 versus 8.3 mths, HR 0.99 32.9 versus 32.6%	0.941 0.787
Nabholtz et al. [15]	Phase 3, randomized, double-blind	353	Anastrozole 1 mg versus tamoxifen 20 mg	TTP OR	11.1 versus 5.6 moths HR, 1.44 21 versus 17%	0.005
Paridaens et al. [16]	Phase 3, randomized, open-label	371	Exemestane 25 mg versus tamoxifen 20 mg	PFS	HR 0.84, 95% CI 0.67–1.05	0.121
Mouridsen et al. [17]	Phase 3, randomized, double-blind	916	Letrozole 2.5 mg versus tamoxifen 20 mg	TTP	9.4 versus 6.0 moths, HR 0.72	<0.0001
PALOMA-1/TRIO-18 [18]	Phase 2, open-label, randomized	165	Letrozole 2.5 mg versus letrozole 2.5 mg + palbociclib 125 mg	PFS	10.2 versus 20.2 mths, HR 0.488	≤0.001
FIRST [19]	Phase 2, open-label, randomized	205	Fulvestrant 500 mg versus anastrozole 1 mg	CBR	72.5 versus 67%	0.386
*Second line*						
BOLERO-2 [20]	Phase 3, randomized, double-blind	724	Everolimus 10 mg + exemestane 25 mg versus exemestane 25 mg	PFS	6.9 versus 2.8 mths, HR 0.43	<0.001
PALOMA3 [21]	Phase 3, randomized, double-blinded	521	Palbociclib 125 mg + fulvestrant 500 mg versus. fulvestrant 500 mg	PFS	9.2 versus 3.8 mths, HR 0.42	<0.001

No, number; TTP, time to progression; OR, objective response; mths, month; HR, hazard ratio; PFS, progression-free survival; CBR, clinical benefit rate (proportion of patients with objective response or stable disease for ≥24 weeks).

## References

[1] DeSantis C. E., Fedewa S. A., Sauer A. G., Kramer J. L., Smith R. A., Jemal A. (2016). Breast cancer statistics, 2015: convergence of incidence rates between black and white women. *CA: A Cancer Journal for Clinicians*.

[2] Siegel R. L., Miller K. D., Jemal A. (2017). Cancer statistics. *Cancer Journal for Clinicians*.

[3] Yancik R. (1997). Cancer burden in the aged: an epidemiologic and demographic overview. *Cancer*.

[4] Berry D. A., Cronin K. A., Plevritis S. K. (2005). Effect of screening and adjuvant therapy on mortality from breast cancer. *The New England Journal of Medicine*.

[5] Cataliotti L., Buzdar A. U., Noguchi S. (2006). Comparison of anastrozole versus tamoxifen as preoperative therapy in postmenopausal women with hormone receptor-positive breast cancer: the Pre-Operative "Arimidex" Compared to Tamoxifen (PROACT) trial. *Cancer*.

[6] Smith I. E., Dowsett M., Ebbs S. R. (2005). Neoadjuvant treatment of postmenopausal breast cancer with anastrozole, tamoxifen, or both in combination: the Immediate Preoperative Anastrozole, Tamoxifen, or Combined With Tamoxifen (IMPACT) multicenter double-blind randomized trial. *Journal of Clinical Oncology*.

[7] Ellis M. J., Suman V. J., Hoog J. (2011). Randomized phase II neoadjuvant comparison between letrozole, anastrozole, and exemestane for postmenopausal women with estrogen receptor-rich stage 2 to 3 breast cancer: clinical and biomarker outcomes and predictive value of the baseline PAM50-based intrinsic subtype—ACOSOG Z1031. *Journal of Clinical Oncology*.

[8] Eiermann W., Paepke S., Appfelstaedt J. (2001). Preoperative treatment of postmenopausal breast cancer patients with letrozole: a randomized double-blind multicenter study. *Annals of Oncology*.

[9] Semiglazov V. F., Semiglazov V. V., Dashyan G. A. (2007). Phase 2 randomized trial of primary endocrine therapy versus chemotherapy in postmenopausal patients with estrogen receptor-positive breast cancer. *Cancer*.

[10] Alba E., Calvo L., Albanell J. (2012). Chemotherapy (CT) and hormonotherapy (HT) as neoadjuvant treatment in luminal breast cancer patients: results from the GEICAM/2006-03, a multicenter, randomized, phase-II study. *Annals of Oncology*.

[11] Cuzick J., Sestak I., Baum M. (2010). Effect of anastrozole and tamoxifen as adjuvant treatment for early-stage breast cancer: 10-year analysis of the ATAC trial. *The Lancet Oncology*.

[12] Group BIGC, Mouridsen H., Giobbie-Hurder A. (2009). Letrozole therapy alone or in sequence with tamoxifen in women with breast cancer. *The New England Journal of Medicine*.

[13] Goss P. E., Ingle J. N., Martino S. (2005). Randomized trial of letrozole following tamoxifen as extended adjuvant therapy in receptor-positive breast cancer: updated findings from NCIC CTG MA.17. *Journal of the National Cancer Institute*.

[14] Bonneterre J., Thürlimann B., Robertson J. F. R. (2000). Anastrozole versus tamoxifen as first-line therapy for advanced breast cancer in 668 postmenopausal women: results of the tamoxifen or arimidex randomized group efficacy and tolerability study. *Journal of Clinical Oncology*.

[15] Nabholtz J. M., Buzdar A., Pollak M. (2000). Anastrozole is superior to tamoxifen as first-line therapy for advanced breast cancer in postmenopausal women: results of a North American multicenter randomized trial. *Journal of Clinical Oncology*.

[16] Paridaens R. J., Dirix L. Y., Beex L. V. (2008). Phase III study comparing exemestane with tamoxifen as first-line hormonal treatment of metastatic breast cancer in postmenopausal women: the european organisation for research and treatment of cancer breast cancer cooperative group. *Journal of Clinical Oncology*.

[17] Mouridsen H., Gershanovich M., Sun Y. (2003). Phase III study of letrozole versus tamoxifen as first-line therapy of advanced breast cancer in postmenopausal women: analysis of survival and update of efficacy from the International Letrozole Breast Cancer Group. *Journal of Clinical Oncology*.

[18] Finn R. S., Crown J. P., Lang I. (2015). The cyclin-dependent kinase 4/6 inhibitor palbociclib in combination with letrozole versus letrozole alone as first-line treatment of oestrogen receptor-positive, HER2-negative, advanced breast cancer (PALOMA-1/TRIO-18): a randomised phase 2 study. *The Lancet Oncology*.

[19] Robertson J. F. R., Llombart-Cussac A., Rolski J. (2009). Activity of fulvestrant 500 mg versus anastrozole 1 mg as first-line treatment for advanced breast cancer: Results from the FIRST study. *Journal of Clinical Oncology*.

[20] Baselga J., Campone M., Piccart M. (2012). Everolimus in postmenopausal hormone-receptor-positive advanced breast cancer. *The New England Journal of Medicine*.

[21] Turner N. C., Ro J., André F. (2015). Palbociclib in hormone-receptor–positive advanced breast cancer. *The New England Journal of Medicine*.

[22] Welch H. G., Albertsen P. C., Nease R. F., Bubolz T. A., Wasson J. H. (1996). Estimating treatment benefits for the elderly: the effect of competing risks. *Annals of Internal Medicine*.

[23] Lichtman S. M. (2004). Chemotherapy in the Elderly. *Seminars in Oncology*.

[24] Schairer C., Mink P. J., Carroll L., Devesa S. S. (2004). Probabilities of death from breast cancer and other causes among female breast cancer patients. *Journal of the National Cancer Institute*.

[25] Land L. H., Dalton S. O., Jensen M.-B., Ewertz M. (2012). Influence of comorbidity on the effect of adjuvant treatment and age in patients with early-stage breast cancer. *British Journal of Cancer*.

[26] Decoster L., Van Puyvelde K., Mohile S. (2015). Screening tools for multidimensional health problems warranting a geriatric assessment in older cancer patients: an update on SIOG recommendations. *Annals of Oncology*.

[27] Walde N. V., Jagsi R., Dotan E. (2016). Nccn guidelines (r) insights older adult oncology, version 2.2016 featured updates to the nccn guidelines. *Journal of the National Comprehensive Cancer Network*.

[28] Clough-Gorr K. M., Thwin S. S., Stuck A. E., Silliman R. A. (2012). Examining five- and ten-year survival in older women with breast cancer using cancer-specific geriatric assessment. *European Journal of Cancer*.

[29] Soubeyran P., Fonck M., Blanc-Bisson C. (2012). Predictors of early death risk in older patients treated with first-line chemotherapy for cancer. *Journal of Clinical Oncology*.

[30] Tesarova P. (2016). Specific aspects of breast cancer therapy of elderly women. *BioMed Research International*.

[31] Fisher C. J., Egan M. K., Smith P., Wicks K., Millis R. R., Fentiman I. S. (1997). Histopathology of breast cancer in relation to age. *British Journal of Cancer*.

[32] Syed B. M., Green A. R., Paish E. C. (2013). Biology of primary breast cancer in older women treated by surgery: with correlation with long-term clinical outcome and comparison with their younger counterparts. *British Journal of Cancer*.

[33] Diab S. G., Elledge R. M., Clark G. M. (2000). Tumor characteristics and clinical outcome of elderly women with breast cancer. *Journal of the National Cancer Institute*.

[34] Poltinnikov I. M., Rudoler S. B., Tymofyeyev Y., Kennedy J., Anne P. R., Curran W. J. (2006). Impact of Her-2 Neu overexpression on outcome of elderly women treated with wide local excision and breast irradiation for early stage breast cancer: an exploratory analysis. *American Journal of Clinical Oncology*.

[35] Owusu C., Lash T. L., Silliman R. A. (2007). Effectiveness of adjuvant tamoxifen therapy among older women with early stage breast cancer. *The Breast Journal*.

[36] Leone J., Leone B. A., Leone J. P. (2016). Adjuvant systemic therapy in older women with breast cancer. *Breast Cancer : Targets and Therapy*.

[37] Singh R., Hellman S., Heimann R. (2004). The natural history of breast carcinoma in the elderly: implications for screening and treatment. *Cancer*.

[38] Olmi P., Fallai C., Cerrotta A. M., Lozza L., Badii D. (2003). Breast cancer in the elderly: the role of adjuvant radiation therapy. *Critical Review in Oncology/Hematology*.

[39] Molino A., Giovannini M., Auriemma A. (2006). Pathological, biological and clinical characteristics, and surgical management, of elderly women with breast cancer. *Critical Review in Oncology/Hematology*.

[40] Dimitrakopoulos F.-I. D., Kottorou A., Antonacopoulou A. G., Makatsoris T., Kalofonos H. P. (2015). Early-stage breast cancer in the elderly: confronting an old clinical problem. *Journal of Breast Cancer*.

[41] Santen R. J., Harvey H. A. (1999). Use of aromatase inhibitors in breast carcinoma. *Endocrine-Related Cancer*.

[42] Smith I. E., Dowsett M. (2003). Aromatase inhibitors in breast cancer. *The New England Journal of Medicine*.

[43] Kudachadkar R., O'Regan R. M. (2005). Aromatase inhibitors as adjuvant therapy for postmenopausal patients with early stage breast cancer. *CA: A Cancer Journal for Clinicians*.

[44] Baum M., Buzdar A. U., Cuzick J. (2002). Anastrozole alone or in combination with tamoxifen versus tamoxifen alone for adjuvant treatment of postmenopausal women with early breast cancer: first results of the ATAC randomised trial. *The Lancet*.

[45] Howell A., Cuzick J., Baum M., Buzdar A., Dowsett M., Forbes J. F. (2005). Results of the atac (arimidex, tamoxifen, alone or in combination) trial after completion of 5 years' adjuvant treatment for breast cancer. *The Lancet*.

[46] Breast International Group 1-98 Collaborative G, Thürlimann B., Keshaviah A. (2005). A comparison of letrozole and tamoxifen in postmenopausal women with early breast cancer. *The New England Journal of Medicine*.

[47] Muss H. B., Tu D., Ingle J. N. (2008). Efficacy, toxicity, and quality of life in older women with early-stage breast cancer treated with letrozole or placebo after 5 years of tamoxifen: NCIC CTG intergroup trial MA.17. *Journal of Clinical Oncology*.

[48] Early Breast Cancer Trialists' Collaborative G (2015). Aromatase inhibitors versus tamoxifen in early breast cancer: patient-level meta-analysis of the randomised trials. *The Lancet*.

[49] Dowsett M., Cuzick J., Ingle J. (2010). Meta-analysis of breast cancer outcomes in adjuvant trials of aromatase inhibitors versus tamoxifen. *Journal of Clinical Oncology*.

[50] Vergote I., Bonneterre J., Thürlimann B. (2000). Randomised study of anastrozole versus tamoxifen as first-line therapy for advanced breast cancer in postmenopausal women. *European Journal of Cancer*.

[51] Mouridsen H., Chaudri-Ross H. A. (2004). Efficacy of first-line letrozole versus tamoxifen as a function of age in postmenopausal women with advanced breast cancer. *The Oncologist*.

[52] Ho A., Dowdy S. F. (2002). Regulation of G1 cell-cycle progression by oncogenes and tumor suppressor genes. *Current Opinion in Genetics & Development*.

[53] Arber N., Doki Y., Han E. K.-H. (1997). Antisense to cyclin D1 inhibits the growth and tumorigenicity of human colon cancer cells. *Cancer Research*.

[54] Sauter E. R., Nesbit M., Litwin S., Klein-Szanto A. J. P., Cheffetz S., Herlyn M. (1999). Antisense cyclin D1 induces apoptosis and tumor shrinkage in human squamous carcinomas. *Cancer Research*.

[55] Finn R. S., Dering J., Conklin D. (2009). PD 0332991, a selective cyclin D kinase 4/6 inhibitor, preferentially inhibits proliferation of luminal estrogen receptor-positive human breast cancer cell lines in vitro. *Breast Cancer Research*.

[56] Finn R. S., Hurvitz S. A., Allison M. A., Applebaum S., Glaspy J. (2009). Phase i study of pd 0332991, a novel, oral, cyclin-d kinase (cdk) 4/6 inhibitor in combination with letrozole, for first-line treatment of metastatic post-menopausal, estrogen receptor-positive (er plus ), human epidermal growth factor receptor 2 (her2)-negative breast cancer. *Cancer Research*.

[57] Wakeling A. E., Dukes M., Bowler J. (1991). A potent specific pure antiestrogen with clinical potential. *Cancer Research*.

[58] Robertson J. F. R., Lindemann J. P. O., Llombart-Cussac A. (2012). Fulvestrant 500 mg versus anastrozole 1 mg for the first-line treatment of advanced breast cancer: Follow-up analysis from the randomized 'FIRST' study. *Breast Cancer Research and Treatment*.

[59] Ellis M. J., Llombart-Cussac A., Feltl D. (2015). Fulvestrant 500 mg versus anastrozole 1 mg for the first-line treatment of advanced breast cancer: overall survival analysis from the phase II first study. *Journal of Clinical Oncology*.

[60] Burstein H. J. (2011). Novel agents and future directions for refractory breast cancer. *Seminars in Oncology*.

[61] Johnston S. R. D., Arteaga C., Winer E., Dowsett M., Kumar R., Come S. (2006). Clinical efforts to combine endocrine agents with targeted therapies against epidermal growth factor receptor/human epidermal growth factor receptor 2 and mammalian target of rapamycin in breast cancer. *Clinical Cancer Research*.

[62] Schiff R., Massarweh S. A., Shou J. (2004). Cross-talk between estrogen receptor and growth factor pathways as a molecular target for overcoming endocrine resistance. *Clinical Cancer Research*.

[63] Yardley D. A., Noguchi S., Pritchard K. I. (2013). Everolimus plus exemestane in postmenopausal patients with HR^+^ breast cancer: BOLERO-2 final progression-free survival analysis. *Advances in Therapy*.

[64] Pritchard K. I., Burris H. A., Ito Y. (2013). Safety and efficacy of everolimus with exemestane vs. Exemestane alone in elderly patients with HER2-negative, hormone receptor-positive breast cancer in BOLERO-2. *Clinical Breast Cancer*.

[65] Coombes R. C., Hall E., Gibson L. J. (2004). A randomized trial of exemestane after two to three years of tamoxifen therapy in postmenopausal women with primary breast cancer. *The New England Journal of Medicine*.

[66] Love R. R., Mazess R. B., Barden H. S. (1992). Effects of tamoxifen on bone mineral density in postmenopausal women with breast cancer. *The New England Journal of Medicine*.

[67] Neuner J. M., Yen T. W., Sparapani R. A., Laud P. W., Nattinger A. B. (2011). Fracture risk and adjuvant hormonal therapy among a population-based cohort of older female breast cancer patients. *Osteoporosis International*.

[68] Becker T., Lipscombe L., Narod S., Simmons C., Anderson G. M., Rochon P. A. (2012). Systematic review of bone health in older women treated with aromatase inhibitors for early-stage breast cancer. *Journal of the American Geriatrics Society*.

[69] Hadji P., Aapro M. S., Body J. J. (2011). Management of aromatase inhibitor-associated bone loss in postmenopausal women with breast cancer: practical guidance for prevention and treatment. *Annals of Oncology*.

[70] Eidtmann H., de Boer R., Bundred N. (2010). Efficacy of zoledronic acid in postmenopausal women with early breast cancer receiving adjuvant letrozole: 36-month results of the ZO-FAST Study. *Annals of Oncology*.

[71] Brufsky A., Harker G., Beck J. (2009). The effect of zoledronic acid on aromatase inhibitor-associated bone loss in postmenopausal women with early breast cancer receiving adjuvant letrozole: the z-fast study 5-year final follow-up. *Cancer Research*.

[72] Llombart A., Frassoldati A., Paija O. (2012). Immediate administration of zoledronic acid reduces aromatase inhibitorassociated bone loss in postmenopausal women with early breast cancer: 12-month analysis of the E-ZO-FAST trial. *Clinical Breast Cancer*.

[73] Hines S. L., Mincey B., Dentchev T. (2009). Immediate versus delayed zoledronic acid for prevention of bone loss in postmenopausal women with breast cancer starting letrozole after tamoxifen-N03CC. *Breast Cancer Research and Treatment*.

[74] Ellis G. K., Bone H. G., Chlebowski R. (2008). Randomized trial of denosumab in patients receiving adjuvant aromatase inhibitors for nonmetastatic breast cancer. *Journal of Clinical Oncology*.

[75] Early Breast Cancer Trialists' Collaborative G (2015). Adjuvant bisphosphonate treatment in early breast cancer: Meta-analyses of individual patient data from randomised trials. *The Lancet*.

[76] Amir E., Seruga B., Niraula S., Carlsson L., Ocaña A. (2011). Toxicity of adjuvant endocrine therapy in postmenopausal breast cancer patients: a systematic review and meta-analysis. *Journal of the National Cancer Institute*.

[77] Baum M., Buzdar A., Cuzick J. (2003). Anastrozole alone or in combination with tamoxifen versus tamoxifen alone for adjuvant treatment of postmenopausal women with early-stage breast cancer: results of the ATAC (Arimidex, Tamoxifen Alone or in Combination) trial efficacy and safety update analyses. *Cancer*.

[78] Crivellari D., Sun Z., Coates A. S. (2008). Letrozole compared with tamoxifen for elderly patients with endocrine-responsive early breast cancer: The BIG 1-98 trial. *Journal of Clinical Oncology*.

[79] Puts M. T. E., Tu H. A., Tourangeau A. (2014). Factors influencing adherence to cancer treatment in older adults with cancer: a systematic review. *Annals of Oncology*.

[80] Kardas P., Lewek P., Matyjaszczyk M. (2013). Determinants of patient adherence: a review of systematic reviews. *Frontiers in Pharmacology*.

[81] Riley G. F., Warren J. L., Harlan L. C., Blackwell S. A. (2011). Endocrine therapy use among elderly hormone receptor-positive breast cancer patients enrolled in Medicare Part D. *Medicare and Medicaid Research Review*.

[82] Ursem C. J., Bosworth H. B., Shelby R. A., Hwang W., Anderson R. T., Kimmick G. G. (2015). Adherence to adjuvant endocrine therapy for breast cancer: importance in women with low income. *Journal of Women's Health*.

[83] Kimmick G., Anderson R., Camacho F., Bhosle M., Hwang W., Balkrishnan R. (2009). Adjuvant hormonal therapy use among insured, low-income women with breast cancer. *Journal of Clinical Oncology*.

[84] Hershman D. L., Kushi L. H., Shao T. (2010). Early discontinuation and nonadherence to adjuvant hormonal therapy in a cohort of 8,769 early-stage breast cancer patients. *Journal of Clinical Oncology*.

